# Effect of finisher Gentlefile Brush, XP-Endo Finisher and Passive Ultrasonic Irrigation in removing radiopaque double antibiotic paste from the root canal

**DOI:** 10.6026/973206300213186

**Published:** 2025-09-30

**Authors:** Rajnandini Jakhetiya, Maulsree Guleria, Gurudutt Nayak, Isha Dixit, Aakanksha Agrawal, Rohan Singh

**Affiliations:** 1 Department of Conservative Dentistry and Endodontics, Mansarovar Dental College, Bhopal, Madhya Pradesh, India

**Keywords:** Double antibiotic paste, Finisher Gentlefile Brush, XP-Endo finisher, passive ultrasonic irrigation, root canal

## Abstract

The complete elimination of double antibiotic paste (DAP) before obturation is a critical clinical challenge in endodontics. This study evaluated three systems for removing radiopaque DAP from root canals. After being instrumented and filled with radiopaque DAP, 42 human mandibular premolars were incubated for 14 days. Specimens were equally divided into three groups and medicament was removed using the Finisher Gentlefile Brush, XP-Endo Finisher and Passive Ultrasonic Irrigation (PUI). The Finisher Gentlefile Brush demonstrated exceptional DAP removal, achieving complete elimination in the middle third and superior cleaning rates in both coronal and apical regions. Despite the Finisher Gentlefile Brush's remarkable performance, complete DAP elimination was not achieved with any method, indicating a persistent clinical challenge.

## Background:

Successful endodontic therapy depends on the effective elimination of bacteria and their toxic by-products from complex root canal systems, which requires the sophisticated integration of mechanical instrumentation with advanced irrigation protocols and strategic intracanal medicament application [1]. While calcium hydroxide [Ca(OH)_2_] has historically dominated as the preferred intracanal agent due to proven antimicrobial efficacy, the polymicrobial nature of root canal pathology necessitates broader antimicrobial strategies [2]. Despite serious clinical drawbacks, such as detrimental effects on stem cells and troublesome tooth discolouration, triple antibiotic paste (TAP), which consists of metronidazole, ciprofloxacin and minocycline, became a widely used intracanal medication [3, 4]. Consequently, the development of DAP, incorporating metronidazole and ciprofloxacin, boosted clinical acceptance by retaining effective antibacterial properties while eliminating discoloration concerns associated with minocycline [5]. DAP has proven to be as effective as TAP in root canals against *Enterococcus faecalis* and it also yields favourable outcomes in underdeveloped teeth following regenerative endodontic procedures [6]. However, a significant challenge for clinicians lies in verifying the proper placement and complete removal of antibiotic medicaments within root canal systems due to their radiographic invisibility. To overcome this, radiopaque agents like barium sulphate (BaSO4) can be incorporated. This allows for radiographic visualization, confirming complete canal filling and subsequent removal, all while preserving the crucial antimicrobial effectiveness and therapeutic properties [7]. One of the most difficult parts of modern endodontic practice is the clinical requirement for total DAP removal before obturation, since leftover medication can seriously impair apical seal integrity and hinder appropriate sealer penetration into dentinal tubules [4]. In contemporary dental practice, there are several techniques employed to eradicate DAP from the root canal, among which PUI and XP-Endo Finisher (FKG Dentaire, La-Chaux-de-Fonds, Switzerland) have proved to be the best alternatives with mixed results [8]. Recently developed, the Gentlefile system, featuring its innovative Finisher Gentlefile Brush (MedicNRG Ltd, Kibbutz Afikim, Israel), utilizes six flexible stainless-steel wire strands that expand outward at high rotational speeds. This creates centrifugal movement and enhanced swirling agitation, optimizing irrigant contact and debris removal from canal walls [9]. Preliminary studies suggest superior performance of this brush in oval canal debridement compared to conventional techniques, indicating potential advantages in complex cleaning scenarios [10, 11]. A comprehensive literature search revealed no published investigations examining the effectiveness of the Finisher Gentlefile Brush in eradicating DAP from root canal systems. In contrast, the XP-Endo Finisher exploits Max-wire nickel-titanium (NiTi) alloy technology, utilizing shape-memory principles. It transitions from a straight, martensitic phase at room temperature to an expanded, curved austenitic phase at body temperature, reaching up to 6 mm in diameter. This design facilitates cleaning of complex morphologies and difficult areas, improves removal of intracanal medicament in curved and oval canals, aids in eliminating root canal filling materials and reduces bacterial load [12]. Similarly, PUI utilizes cavitation and acoustic streaming mechanisms through oscillating ultrasonic tips to enhance cleaning efficacy [13, 14]. Several devices are available for PUI, among which the most recently developed Ultra-X ultrasonic device (Eighteenth, Jiangsu Province, China) has been commonly used. Despite these advancements, complete DAP removal remains challenging, particularly in oval canals with limited access due to circular preparations [15, 16]. Therefore, it is of interest to evaluate the comparative effectiveness of Finisher Gentlefile Brush, XP-Endo Finisher and PUI for eliminating radiopaque double antibiotic paste from root canals.

## Methods and Materials:

## Sample size calculation and standardization:

Sample size calculation was determined using G*Power software (Version 3.1.9.4, Heinrich-Heine, Dusseldorf, Germany) with an effect size of 0.8, alpha of 0.05 and 80% power, yielding 14 specimens per experimental group. The study used 42 recently removed human mandibular premolars with curvature <5, single root canal, fully formed apex, single apical foramen, no signs of internal resorption, no pulp stones, root canal calcification, obstruction, or previous root canal therapy. Teeth were stored in 3% sodium hypochlorite (NaOCl) for 48 hours, scaled ultrasonically and conserved in 10% formalin till used. Roots were decoronated to a customized length of 16±1 mm using diamond discs. The working length (WL) was determined to be 1 mm less than the length at which, when viewed under a dental operating microscope (DOM) at x25 magnification, a size 10 K-file (MANI, INC., Togachi, Japan) was visible at the apical foramen. Teeth were chosen using apical gauging, maintaining the initial K-file's maximum size at ≤ 1 mm of the apex at size 20.

## Preparations of radiopaque DAP:

Radiopaque DAP was prepared by dissolving 50 mg each of metronidazole (Unichem Laboratories Ltd., India) and ciprofloxacin (Research Lab Chemical Corporation, India) in 10 mL sterile water, yielding 5 mg/mL solutions. Subsequently, 3g BaSO4 was then added with vigorous stirring to create a radiopaque DAP slurry. To get the ideal pasty consistency, 0.7g of methyl cellulose powder was then dissolved at ambient temperature. To create a homogenous injectable paste free of bubbles, the final radiopaque DAP was centrifuged for 15 minutes at 7000 rpm (DLAB Scientific Co., Ltd., China).

## Root canal instrumentation:

Canal preparation utilized ProTaper Gold Rotary systems (Dentsply Sirona, Ballaigues, Switzerland) with a crown-down technique, to achieve final preparation to F5 size (#50/0.05 taper). Canals were copiously irrigated with 5mL of 3% NaOCl (Prevest Denpro Ltd., Jammu, India) after each instrument sequence using 30-gauge side-vented needles (RC Twents, Prime Dental Products Pvt. Ltd.). Final irrigation involved sequential application of 5mL 17% ethylene diamine tetraacetic acid (EDTA) (Prevest DenPro Ltd., Jammu, India), then by 5mL normal saline (Abaris Healthcare Pvt. Ltd., Mehsana, Gujarat, India), followed by drying with sterile absorbent points (Dentsply Sirona, Ballaigues, Switzerland).

## DAP placement and incubation:

DAP was delivered into root canals via a syringe delivery system with radiographic verification. Coronal portions were restored with interim restorative material (Cavit G 3M ESPE Dental Products). Specimens were then incubated at 37°C with 100% humidity for fourteen days in controlled incubators (ACMAS Technologies INC., Delhi, India).

## DAP removal protocol:

Following incubation, temporary fillings were removed and #15 K-files were inserted to WL and instrumented to loosen DAP material. All specimens' canals were foremost irrigated with 5 ml of 3% NaOCl using a closed-end side-vented 30-gauge needle (Septodont Healthcare India Pvt. Ltd., Maharashtra, India) in a syringe that was positioned 1 mm short of the WL at a flow rate of 5 ml/min. Subsequently, samples were subjected to random allocation into 3 investigational groups (n = 14):

## Group 1:

## Finisher Gentlefile Brush:

The irrigant activation protocol utilized a Finisher Gentlefile Brush (0.25 mm tip diameter) positioned 1 mm coronal to the WL. The brush was operated at 6500 rpm with 7-8 mm amplitude vertical strokes for 60 seconds to enhance solution mixing. Subsequently, 5 mL of 17% EDTA was introduced and activated using identical parameters for an additional 60 seconds. The treatment sequence concluded with copious irrigation using 5 mL of normal saline. Each specimen was treated with a new Finisher Gentlefile Brush.

## Group 2:

## XP-Endo Finisher:

XP-Endo Finisher files (0.25 mm) were operated using an X-Smart endomotor (Dentsply Maillefer, Ballaigues, Switzerland) at 800 rpm/1 Ncm torque. After adjusting the file length with a rubber stopper in its plastic sleeve, it was straightened using Roeko Endo-Frost spray (Coltene-Whaledent, Germany) before removal. A slight lateral movement was then used to extract the file from the plastic tube. The file was inserted 1 mm short of the WL and operated with gentle vertical movements of 7-8 mm amplitude for 1 minute. Following irrigation with 5 mL of 17% EDTA, the activation procedure was repeated for another minute. The treatment concluded with a final saline flush of 5 mL. Each specimen was treated with a new XP-Endo Finisher file.

## Group 3:

## PUI:

Ultrasonic activation was executed using an Ultra X ultrasonic device equipped with a #25/0.02 Ultra X tip. The tip was placed 1 mg above the WL and the irrigant was ultrasonically activated for 1 minute. After infusing 5 mL of 17% EDTA, ultrasonic agitation was carried out once again for one minute with the same tip positioning. Each specimen treatment concluded with a 5 mL normal saline rinse. Each Ultra X tip was utilized for three consecutive specimens.

## Assessments of the remaining radiopaque DAP:

Following irrigation, root canals were dried using absorbent paper points. Longitudinal grooves were prepared on the buccal and lingual surfaces at the root's maximum buccolingual width using a diamond disc under copious water cooling, after which the teeth were carefully split into halves using a chisel and mallet. The root half displaying the visible semi-canal lumen with greater DAP remnants was selected and positioned on mm graph paper to delineate coronal, middle and apical thirds. Digital images were obtained using a Canon EOS1300D camera (Canon Inc., Taiwan) coupled to a DOM at 25x magnification, with specimens coded to ensure examiner blinding. Two calibrated examiners, unaware of treatment groups, scored residual DAP using van der Sluis *et al.*'s classification [13]: score "0"-canal surface free of DAP, score "1"-less than half the surface filled with DAP, score "2"-more than half the surface filled with DAP and score "3"-surface filled with DAP (Figure 1 - see PDF). DAP remnants received separate evaluation; scoring and documentation across all canal thirds in selected specimen halves. When scoring discrepancies arose between examiners, cases were re-evaluated through collaborative discussion until consensus was achieved.

## Statistical analysis:

Data analysis utilized SPSS software for Windows, version 25.0 (IBM SPSS Inc., Chicago, IL, USA). The effectiveness of different DAP removal methods was expressed in percentages. Chi-square tests were used to compare categorical variables and a p-value of < .05 was considered significant.

## Results:

Inter-examiner agreement was strong; however, no system achieved complete DAP removal across all regions. Figure 2 (see PDF) shows the score distribution across canal thirds. Intergroup comparisons revealed significant differences (p>0.05), with the Finisher Gentlefile Brush outperforming both XP-Endo Finisher and PUI in all thirds (p<0.05). PUI was also significantly better than XP-Endo Finisher in the middle and apical thirds (p<0.05). Regarding intragroup analysis, the Finisher Gentlefile Brush exhibited no significant differences across thirds (p>0.05), while the XP-Endo Finisher had significant intragroup variation (p<0.05) and PUI displayed moderate performance with declining efficacy across canal thirds (p>0.05). Overall, the Finisher Gentlefile Brush was the most effective and consistent method, followed by PUI, with the XP-Endo Finisher being the least effective, especially in deeper regions (Table 1 - see PDF).

## Discussion:

Complete debridement and cleaning of the endodontic pulp area, including the elimination of inflammatory mediators and pathogenic flora, is the main objective of root canal therapy. This involves mechanical instrumentation, irrigation protocols and intracanal medicaments [17]. Although Ca(OH)_2_ has been a dominant intracanal agent due to its antimicrobial effectiveness against common endodontic bacteria [2]. The polymicrobial nature of root canal infections often necessitates the use of various antibiotic combinations. The emergence of TAP was a significant milestone in intracanal medicaments, demonstrating remarkable antimicrobial efficacy against diverse bacteria in endodontic infections [1, 17]. However, clinical use revealed limitations, including deleterious effects on stem cells of the apical papilla in regenerative endodontic procedures [3] and severe tooth discoloration [4]. These issues prompted the development of DAP by removing minocycline, the component responsible for the discoloration [5]. The transition to DAP represented a paradigm shift in intracanal medicament therapy. Studies show that DAP maintains effective antibacterial properties and is now preferred over TAP for canal disinfection [4, 6]. Intracanal medicaments critically need broad-spectrum antimicrobial efficacy both against aerobic and anaerobic pathogens, especially resistant ones like *E. faecalis*, which cause treatment failures [2, 6]. DAP excels at eradicating bacteria, particularly in retreatment cases with *E. faecalis* contamination [5]. A significant benefit is the adherence of these antibiotics to the dentin matrix, prolonging their antimicrobial efficacy even after removal [18]. However, a challenge with antibiotic medicaments like DAP is their radiographic invisibility, making it difficult for clinicians to confirm complete penetration. To address this, our study incorporated methylcellulose and BaSO_4_ as a stabilizing polymer and radiopacifier. Previous research indicates that both component has adverse biological effects [7] and Wu *et al.* found that a methylcellulose-BaSO_4_ DAP formulation had no detrimental impact on dental pulp stem cell proliferation and performed comparably even without an opacifier [19]. The thorough eradication of DAP after use is mandatory for a fluid-tight seal during obturation, as its retention in dentinal tubules can block sealer adhesion, leading to microbial percolation [20]. High concentrations of DAP are cytotoxic to stem cells and fibroblasts, inhibiting healing and root maturation [21]. Persistent low concentrations can lead to antibiotic resistance and prolonged exposure can soften dentin, increasing fracture risk [22]. Therefore, removing the paste restores a more stable canal environment before final treatment.

The quest for effective DAP eradication from root canals has led to numerous investigations using diverse methods like hand instruments, rotary systems, syringe irrigation, manual dynamic agitation, ultrasonic/sonic activation, CanalBrush and laser techniques [4, 21]. These methods are often combined with various irrigation solutions such as 17% EDTA, 3% NaOCl, 10% citric acid, 9% 1-hydroxyethylidene-1,1-bisphosphonate (HEBP), 0.5% peracetic acid and 2% chlorhexidine [22, 23, 24- 25]. While PUI and XP-Endo Finisher show superior efficacy for DAP removal, neither has achieved complete elimination from the root canal system [26, 27]. Our investigation scrutinized the efficacy of PUI and XP-Endo Finisher against the novel Finisher Gentlefile brush for eradicating radiopaque DAP from root canals. The Finisher Gentlefile system is an innovative irrigation activation technology featuring an automated handpiece operating at 6500 rpm with a brush that expands to contact the full canal diameter, enhancing solution activation and thorough removal [9, 10]. Prior literature on this system is limited, with only one study comparing its efficacy in Ca(OH)_2_ removal [11], making our study focus on DAP removal particularly significant. Our findings demonstrated near-complete DAP elimination from the apical and middle thirds of the canal, with adequate removal from the coronal region. Residual DAP in the coronal region may be attributed to the larger coronal surface area in mandibular premolars and the adherence of constituents to dentin. Statistical analysis revealed significant differences, confirming the superior efficacy of the Finisher Gentlefile Brush over both PUI and XP-Endo Finisher across all canal thirds. These results align with Pawar *et al.* [11], who also found the Finisher Gentlefile Brush superior for removing oil-based Ca(OH)_2_ paste, validating its mechanical design advantages across different medicaments. Neelakantan *et al.* [10] further supported the Finisher Gentlefile Brush's efficacy, showing that it achieved the least remnant pulp tissue compared to conventional irrigation. The Finisher Gentlefile Brush exhibited excellent efficiency, with the majority of specimens achieving complete removal. The XP-Endo Finisher, a single-file NiTi instrument, is frequently used for intracanal medicament removal. Its exceptional flexibility and unique spoon shape, adopted at body temperature, enable three-dimensional adaptation to canal morphology and navigation into intricate areas [12]. In our evaluation, the XP-Endo Finisher left considerable DAP residue in the apical area, while demonstrating superior performance to PUI in the coronal third with decreased effectiveness in the middle regions. These findings contrast with Gokturk *et al.* [15], who reported superior Ca(OH)_2_ elimination using XP-Endo Finisher in apical areas and documented enhanced apical effectiveness. These outcomes differ from Gawdat *et al.* [28], whose research indicated similar effectiveness between XP-Endo Finisher and PUI for antibiotic paste removal. Although the XP-Endo Finisher performed less well in the middle and apical thirds than PUI, our investigation found that it was as successful in coronal segments; still, total canal clearance was not attained in every area. PUI has been employed as the reference standard for DAP removal due to its established efficacy [13, 15 and 16]. PUI produces acoustic microstreaming and cavitation effects by transmitting energy to the irrigation solution from an ultrasonically vibrating device [13, 14]. Using the Ultra X device at 45 kHz, PUI demonstrated moderate and uniform efficacy across canal thirds, with a slight trend of decreasing complete removal toward the apex. Our findings indicate that PUI has superior efficacy in medicament removal from the apical third compared to the XP-Endo Finisher, aligning with Donnermeyer *et al.* [29] and Arslan *et al.* [25] showed PUI's superiority over XP-Endo Finisher and conventional syringe irrigation for medicament removal. However, in contrast with Türkaydın *et al.* [30], found that the XP-Endo Finisher outperformed PUI for TAP removal. Despite literature contradictions, our study demonstrated PUI's superior performance compared with the XP-Endo Finisher for DAP elimination from the root canals.

In summary, the Finisher Gentlefile Brush proved most effective at removing radiopaque DAP across all root canal levels, demonstrating high efficacy and minimal variation, in contrast, the XP-Endo Finisher showed a significant decline in cleaning ability in the middle and apical thirds, resulting in the least effective performance overall; meanwhile, PUI offered moderate, consistent efficacy that slightly decreased from coronal to apical thirds. Although all systems performed comparably in the coronal third, the Finisher Gentlefile Brush significantly outperformed both XP-Endo Finisher and PUI in the middle and apical regions, establishing it as the most reliable system for debriding intracanal medicaments like DAP. This study assessed remaining intracanal medicaments using a 4-grade scoring system, which is easy to use, repeatable and has high intra-examiner agreement. While previous studies have used techniques like digital photography, stereomicroscopy, scanning electron microscopy (SEM) and micro-computed tomography (micro-CT), this scoring system's limitation is that it only evaluates the surface layer, potentially hindering a comprehensive 3D understanding of removal depth. For more accurate results, volumetric analysis with micro-CT and scanning electron microscopy would be beneficial.

## Conclusion:

The clinical implications of our findings extend beyond simple technique comparison. Despite these technological advances, complete DAP elimination from root canal systems remains challenging. Among the various systems tested, the Finisher Gentlefile Brush proved to be the most effective technique for the removal of radiopaque DAP compared to XP-Endo Finisher and PUI. However, due to the complex nature of medicament-dentin interactions, none could suffice the primary objective of complete removal. Future investigations should examine the efficacy of the Finisher Gentlefile Brush when used in combination with alternative dissolving solutions to achieve complete removal, especially in curved canal systems.
